# Use of a Rigid Curved Laryngoscope for Removal of a Fish Bone in the Hypopharynx

**DOI:** 10.1155/2016/9689521

**Published:** 2016-07-27

**Authors:** Hiroshi Sakaida, Kazuki Chiyonobu, Hajime Ishinaga, Kazuhiko Takeuchi

**Affiliations:** Department of Otorhinolaryngology, Head & Neck Surgery, Mie University Graduate School of Medicine, 2-174 Edobashi, Tsu, Mie 514-8507, Japan

## Abstract

Foreign body ingestion is a commonly encountered clinical problem. In particular, sharp foreign bodies lodged in the esophagus or hypopharynx can cause complications and require urgent removal. Removal by flexible esophagogastroduodenoscopy or rigid esophagoscopy is the treatment of choice and has high success rates, but cases in which these methods are unsuccessful must be treated with an external incision. A 62-year-old man was referred for a fish bone lodged in the hypopharynx that could not be removed by flexible esophagogastroduodenoscopy. We removed the bone transorally using a specially designed rigid curved laryngoscope. Based on our experience, this method may have clear practical value due to advantages of a wide field of view and use of multiple rigid forceps. Indications may be limited, but this novel method may reduce the limitations of noninvasive removal of foreign bodies.

## 1. Introduction

Foreign body ingestion is a common clinical problem for which management varies based on the nature and location of the foreign body and the age and size of the patient. In cases involving sharp objects such as fish or chicken bones or dental dentures in the hypopharynx or more distally in the esophagus, urgent removal is required because of the risk of complications [1, 2]. The American Society for Gastrointestinal Endoscopy (ASGE) issued guidelines entitled “Management of Ingested Foreign Bodies and Food Impactions” that recommend flexible esophagogastroduodenoscopy (EGD) [3]. This method has a high success rate with a low complication rate [4, 5].

For proximal foreign bodies impacted at the level of the upper esophageal sphincter or hypopharyngeal region, rigid esophagoscopy may be helpful [3]. However, if the object cannot be removed using these methods, removal through an external incision is indicated [2, 6–8]. Generally, an endoscopic approach is less invasive than an external incision and facilitates patient recovery following the removal procedure. Reducing the limitations of the endoscopic approach will thus be beneficial for patients. Here, we report a case in which we used a specially designed rigid curved laryngoscope to remove a hypopharyngeal fish bone that could not be removed using flexible EGD. To the best of our knowledge, this is the first report of a case in which a foreign body was removed with this instrument.

## 2. Case Report 

**Figure 1:**
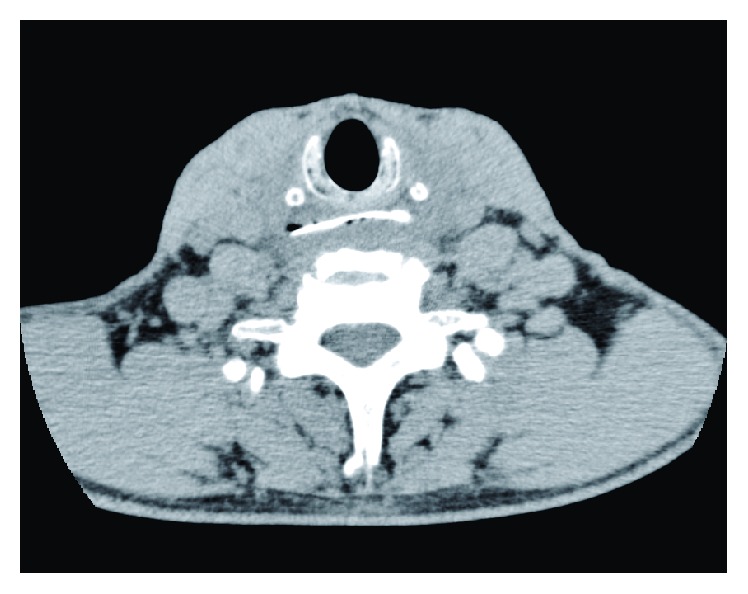
Noncontrast axial computed tomography showing a fish bone measuring 37 mm in length, lodged horizontally at the level of the lower end of the cricoid cartilage. No findings suggestive of mucosal perforation or soft tissue edema around the esophagus are apparent.

A 62-year-old man was referred to our department following ingestion of a fish bone. Four days prior to the referral, the patient had eaten rice cooked with fish (red seabream). Shortly after eating, he felt discomfort, as if a fish bone was stuck in his throat. He visited an otolaryngologist, but a laryngeal fiberscopic examination was unable to detect a fish bone in the larynx or pharynx. However, sore throat and dysphagia deteriorated, and he was examined by another otolaryngologist. Computed tomography revealed a 37 mm long, 8 mm wide fish bone stuck in the hypopharynx (Figure 1). Physicians attempted to remove the fish bone by flexible EGD, but the bone was too tightly stuck to be removed. Eventually, he was referred to our department for further treatment.

**Figure 2:**
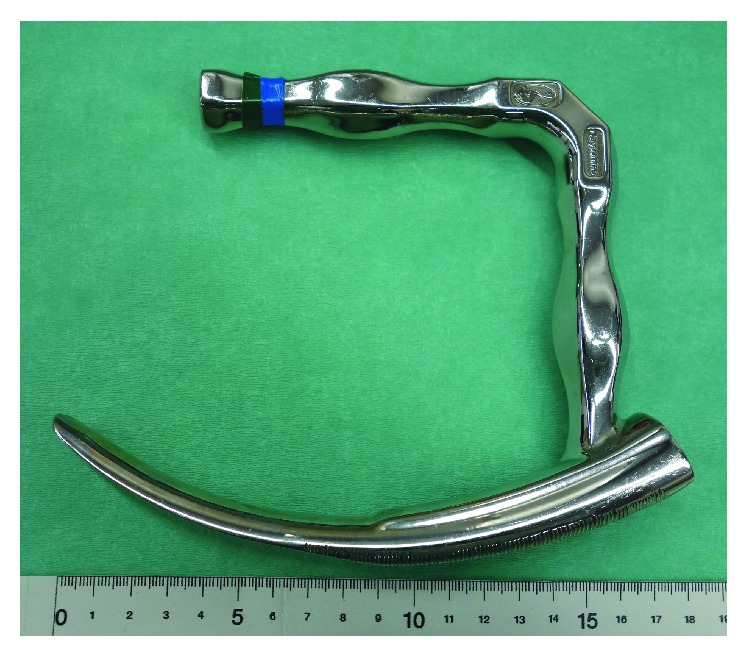
Rigid curved laryngoscope comprising a handle and a blade. The blade is curved along its entire length.

**Figure 3:**
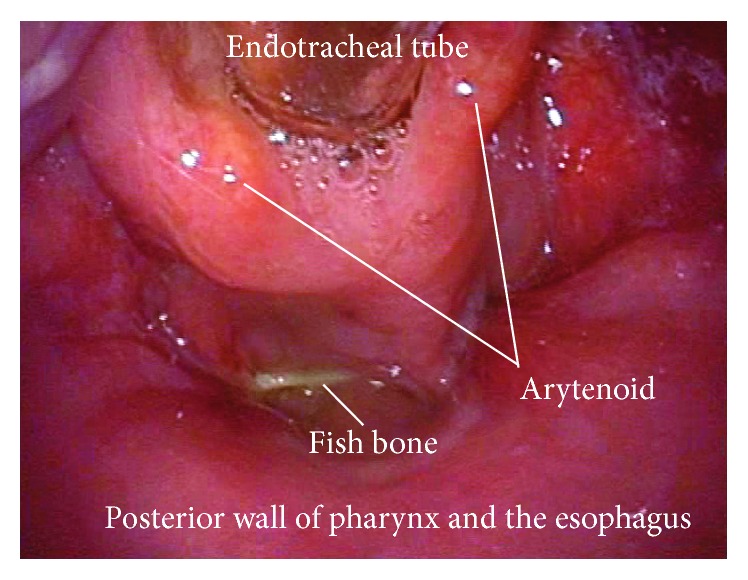
Intraoperative view of the hypopharynx and cervical esophagus. The hypopharynx and cervical esophagus are widely exposed as a continuous cavity. The fish bone can be seen in the hypopharynx with both ends stuck in the mucosa.

**Figure 4:**
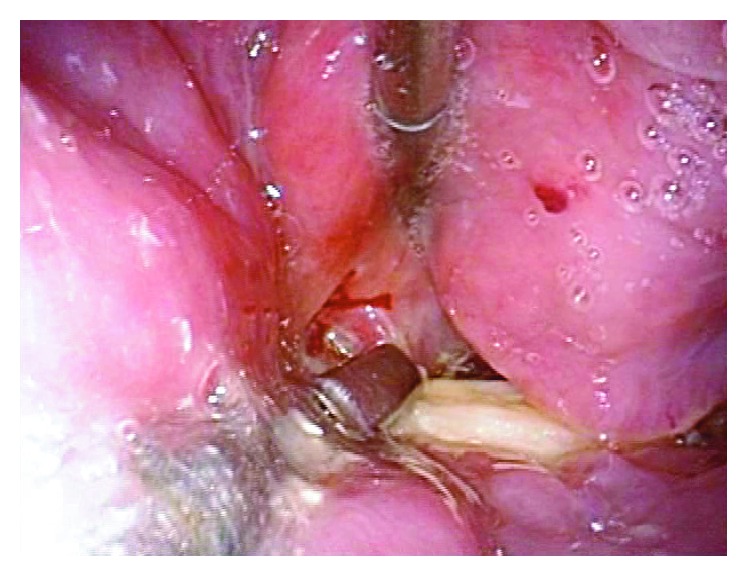
Close-up view of the fish bone during removal. The bone is firmly grasped with rigid forceps, while another pair of forceps applies countertraction to the surrounding mucosa to facilitate atraumatic release of the bone.

**Figure 5:**
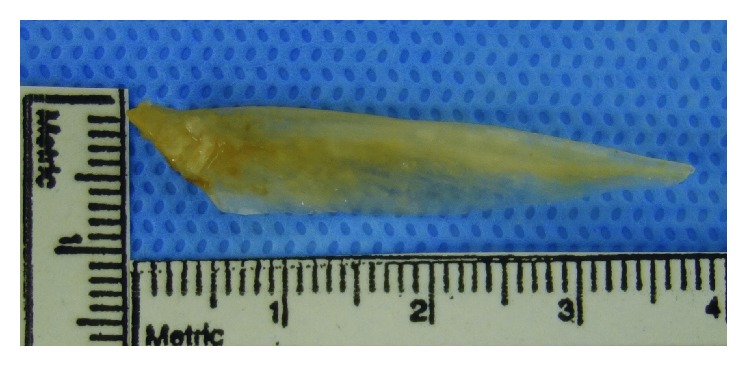
The removed fish bone, 37 mm long and 8 mm wide.

The patient was taken to the operating room. One operator and two assistants performed an emergency operation to remove the fish bone using a rigid curved laryngoscope (Nagashima Medical Instruments Company, Ltd., Tokyo, Japan) (Figure 2). Under general anesthesia with endotracheal intubation, the instrument was inserted into the pharynx. The blade was applied to the laryngeal aspect of the epiglottis. The instrument was lifted forward, exposing both the hypopharynx and cervical esophagus widely. An assistant inserted a flexible laryngeal videoscope for observation and the fish bone was easily identified (Figure 3). The operator firmly grasped the fish bone with rigid forceps but could not move it because both ends were tightly stuck in the esophageal mucosa. Another assistant then applied countertraction to the mucosa with another pair of rigid forceps, so that the operator could pull one of the ends of the fish bone from the mucosa without damaging the mucosa (Figure 4). Eventually, the fish bone was mobilized and removed atraumatically (Figure 5). The patient recovered uneventfully and was discharged after 6 days of observation.

## 3. Discussion

We have described a novel method using a rigid curved laryngoscope to remove a foreign body lodged in the hypopharynx. This instrument was originally developed and has been formally approved as a medical device in Japan. The instrument was designed for endoscopic laryngopharyngeal surgery (ELPS), in which early-stage hypopharyngeal carcinoma is resected transorally under endoscopic vision [9]. The body of the instrument comprises a handle and a blade that is gently curved across the entire length. This instrument is used under general anesthesia. The blade is inserted into the pharynx and applied to the laryngeal aspect of the epiglottis. By lifting the instrument forward, the larynx is lifted, exposing the lumen of both the hypopharynx and proximal cervical esophagus together as a continuous cavity. After exposing the hypopharynx, the handle is firmly attached to a holder that is fixed to the operating table, so that the rigid curved laryngoscope can be suspended in a stable position during the procedure. The widely exposed lumen allows endoscopic resection of a tumor with multiple rigid forceps. More importantly, the multiple forceps can be manipulated individually and independently of the endoscope, allowing more complicated procedures to be performed.

Based on our experience, this method offers several advantages over other methods for removal of sharp objects lodged in the hypopharynx or cervical esophagus. First, the instrument can expose the lumen of the hypopharynx and cervical esophagus together as a continuous cavity. This allows full visualization of foreign bodies lodged in this region. Second, a widely exposed lumen allows the use of rigid forceps such as Magill forceps and those used in laparoscopic surgery. Laparoscopic surgical instruments are used in videolaryngoscopic transoral surgery [10]. Rigid forceps are strong enough to grasp an object firmly without possible accidental release during the procedure. Various forceps, graspers, baskets, snares, and nets are available for use through the working port of a flexible EGD, but these do not seem to be as strong as rigid forceps. In addition, rigid forceps can be manipulated independently of the endoscope, allowing performance of more complicated procedures. Third, an additional set of rigid forceps can be introduced, facilitating application of countertraction and making procedures easier and safer. An object can be grasped and released from the mucosa with one pair of forceps while exerting countertraction on the mucosa with another pair. This type of procedure is analogous with the concept of the “four hands technique” employed in various endoscopic surgeries. Finally, an expanded lumen can prevent direct contact between a sharp object and the mucosa, allowing objects to be removed from the body without scratching the mucosa.

A laryngoscope with interchangeable blades, such as the Macintosh curved blade or the Miller straight blade, is useful for hypopharyngeal foreign bodies that can be easily retrieved with a single pair of forceps. However, when lodged foreign bodies are not easily retrieved, wider exposure of the hypopharynx and stable fixation of the laryngoscope during the procedure are necessary to achieve successful removal. In this situation, the rigid curved laryngoscope is useful because it is designed to widely expose the whole hypopharynx and it has a handle that is attached to a holder fixed to the operation table.

Patient selection is critical for success of the new method. Foreign bodies lodged in the hypopharynx or at the first constriction of the esophagus may be an appropriate indication. Since the instrument is unable to expose the distal part of the cervical esophagus and the thoracic esophagus, removal of foreign bodies lodged distal to the cervical esophagus may not be feasible. Patients with foreign bodies in the hypopharynx or cervical esophagus that cannot be removed by flexible EGD may benefit from this method. Although the indications for the method may be limited, this approach has clear practical value and should play a complementary role with existing methods, reducing the limitations on noninvasive removal of foreign bodies. Further experience is necessary to confirm the utility and specific indications of the method.

In conclusion, in the case reported here, a fish bone tightly stuck in the hypopharynx was removed transorally using a rigid curved laryngoscope. This method seems to have clear practical value due to the wide field of view and the ability to use multiple rigid forceps. Despite the limited indication, this method may reduce limitations on noninvasive removal of foreign bodies.
